# Laparoscopic resection of an incidental retroperitoneal schwannoma in a 74-year-old male: a case report and literature review

**DOI:** 10.3389/fonc.2025.1668644

**Published:** 2025-11-25

**Authors:** Yiwei Hou, Yu Yang, Beihan Li, Ru Zheng, Xiangyu Liao, Qiuyi Peng, Wufei Zhu, Rongchun Xing

**Affiliations:** 1 The First College of Clinical Medical Science, China Three Gorges University, Yichang, Hubei, China; 2 Department of Endocrinology, Yichang Central People’s Hospital, Yichang, Hubei, China; 3 Department of Hepatobiliary Surgery, Yichang Central People’s Hospital, Yichang, Hubei, China; 4 Basic Medical College, Qiqihar Medical University, Qiqihar, Heilongjiang, China; 5 Department of Oncology, Yichang Central People’s Hospital, Yichang, Hubei, China; 6 The Third Affiliated Hospital, Qiqihar Medical University, Qiqihar, Heilongjiang, China

**Keywords:** retroperitoneal schwannoma, laparoscopic resection, hemorrhagic and cystic degeneration, long-term surveillance, case report

## Abstract

**Background:**

This case report highlights a rare retroperitoneal schwannoma in a 74-year-old male, discovered incidentally during a routine physical exam. Schwannomas in the retroperitoneum are uncommon and often present unique diagnostic and management challenges.

**Case summary:**

The patient, a 74-year-old male with hypertension, presented with an incidental finding of a retroperitoneal mass (3.0 x 2.5 cm) on abdominal CT. Further imaging and histopathology confirmed a schwannoma. A laparoscopic resection was performed with no major complications, and the mass was confirmed to be benign with hemorrhagic and cystic degeneration. The patient recovered uneventfully post-surgery and was discharged with recommendations for long-term monitoring.

**Conclusion:**

This case underscores the value of early detection through imaging and the effectiveness of minimally invasive surgery for retroperitoneal.

## 1 Introduction

This case presents a rare and clinically intriguing instance of a retroperitoneal schwannoma in a 74-year-old male, discovered incidentally during a routine physical examination. Schwannomas, while common in peripheral nerves, are infrequently found in the retroperitoneal space, and this case emphasizes the diagnostic and management challenges associated with such rare tumors (1). The primary aim of this case report is to highlight the clinical course, diagnostic approach, and surgical intervention required to manage a retroperitoneal schwannoma in an elderly patient. This case is significant not only due to the rarity of the tumor but also because it underscores the importance of regular imaging and careful surgical planning to minimize risks, especially in patients with multiple comorbidities (2).

A thorough literature review was conducted to explore the clinical presentation, diagnostic workup, and management of retroperitoneal schwannomas. Key search terms included “retroperitoneal schwannoma,” “neurogenic tumor,” and “laparoscopic resection of schwannomas.” (**Table 1**) The case was analyzed in the context of existing literature, which supports the view that retroperitoneal schwannomas often remain asymptomatic until reaching a significant size, at which point they may cause compressive symptoms (3). This report aims to provide valuable insights into the diagnosis, management, and postoperative care of such rare tumors, with the hope of contributing to the broader understanding of their clinical handling.

**Table 1:** Comparative summary of published case reports on retroperitoneal schwannomas and key differences from the index case.

Publication year	Title	Key findings	Differences from this case
2009	Endoscopic resection of a retroperitoneal schwannoma: case report (18).	Describes endoscopic resection of retroperitoneal schwannoma in a 35-year-old man.	Different in surgical method (endoscopic vs. laparoscopic).
2017	Unusual presentation of retroperitoneal Schwannoma: case report (19).	Retroperitoneal schwannoma presenting with vague symptoms, successfully excised with minimal complications.	Similar presentation with benign tumor excised with no recurrence
2020	Laparoscopic Resection of a Large Retroperitoneal Schwannoma: A Case Report (3).	65-year-old male. Symptomatic (abdominal discomfort) 8 cm retroperitoneal schwannoma with cystic degeneration. Laparoscopic resection performed successfully. Benign diagnosis.	Presentation: Symptomatic discovery vs incidental. Tumor Size: Significantly larger (8 cm vs 3 cm). Age: Younger patient
2025	Uncommon retroperitoneal mass in a young adult: A rare case report of retroperitoneal schwannoma and review of diagnostic challenges (20).	A rare presentation of retroperitoneal schwannoma in a young adult, successfully excised.	Similar in tumor excision but highlights rarity in young patients
2025	Malignant retroperitoneal schwannoma in a young adult: rapid recurrence, metastasis, and treatment reflections-a case report (21).	Describes rapid recurrence and metastasis of a malignant retroperitoneal schwannoma.	This case presents malignancy and recurrence, whereas our case describes a benign case with no recurrence

This table chronologically summarizes case reports (2001–2025) highlighting diagnostic and therapeutic approaches for pancreatic neuroendocrine tumors (NETs). Key abbreviations: NEC (neuroendocrine carcinoma), NTRK3 (neurotrophic tyrosine receptor kinase 3). Cases were selected based on relevance to pancreatic NET recurrence, biliary obstruction, and multimodal management (e.g., targeted therapies, neoadjuvant chemotherapy, surgical resection).

## 2 Case presentation

The patient is a 74-year-old male with a chief complaint of an abdominal mass discovered four days prior. He underwent a routine physical examination on May 20, 2025, where an abdominal CT revealed a right retroperitoneal mass (3.0 x 2.5 cm). Additional imaging, including an abdominal ultrasound, indicated multiple hepatic cysts, mild fatty liver, and prostate enlargement with calcifications. No significant symptoms such as abdominal pain, chest discomfort, or urinary disturbances were noted. He had no history of smoking, alcohol consumption, or drug use. He had been managing long-term hypertension with valsartan, and he denied any significant family history of hereditary diseases.

Upon admission, his vital signs were stable, with a temperature of 36.4°C, pulse of 88 beats per minute, blood pressure of 138/85 mmHg, and respiration at 18 breaths per minute. The physical examination was unremarkable, with no jaundice, edema, or abdominal tenderness. Previous surgical history included a cholecystectomy and right renal cyst removal. Routine laboratory tests, including liver function tests, revealed mild elevations in transaminases and bilirubin, which were monitored during the hospital stay.

**Figure 1:**
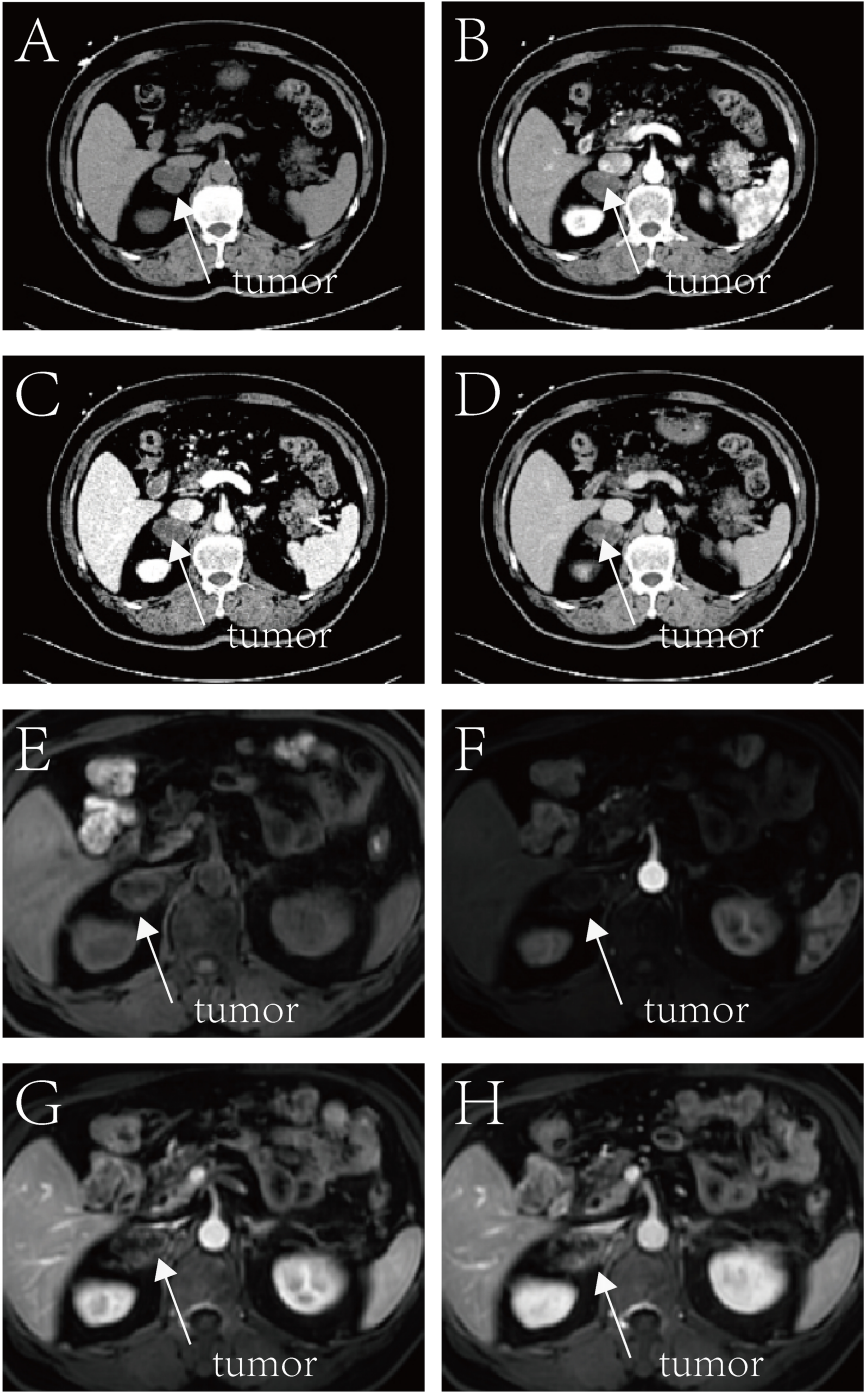
Liver plain scan enhanced hepatic arterial angiography (CTA), portal vein angiography (CTV)CT examination and plain scan enhanced MR Examination. CT **(A–D)** and MR **(E–H)** images demonstrate a mass lesion located in the right retroperitoneal region (arrows). CT imaging reveals a well-defined, heterogeneous, hypodense mass measuring approximately 2.7 × 3.7 cm, with punctate calcifications and clear demarcation from adjacent renal and adrenal structures. The lesion shows marked peripheral heterogeneous enhancement post-contrast administration. MR imaging depicts the lesion as a heterogeneous mass with mixed hyperintense signals on T1-weighted images and mixed hyper-/hypointense signals on T2-weighted images, measuring approximately 2.5 × 3.5 cm, exhibiting prominent peripheral enhancement upon contrast. Imaging characteristics suggest a neurogenic tumor, most likely schwannoma. Additionally, multiple hepatic and left renal cysts are identified, and fatty infiltration of the pancreas is evident.

A comprehensive diagnostic workup included further imaging: a contrast-enhanced CT scan confirmed the retroperitoneal mass, suspected to be a neurogenic tumor, possibly a schwannoma (**Figure 1**). The patient also underwent coronary CT angiography, which identified coronary artery narrowing and myocardial bridging, as well as a coronary artery variant with a 20% stenosis in the left main coronary artery.

The possibility of a benign schwannoma was considered high; however, malignancy could not be entirely ruled out. Liver plain and contrast-enhanced CTA/CTV revealed a right retroperitoneal mass with low and heterogeneous density. Correspondingly, MRI of the hepatobiliary system demonstrated a patchy lesion in the right retroperitoneal region, showing long T1 and long/slightly short T2 mixed signals (**Figure 1**). The imaging findings indicated a cystic-solid lesion with complex internal components, including solid and cystic areas, and the potential presence of hemorrhage or necrosis. Because percutaneous biopsy carried a significant risk of tumor rupture and bleeding, as well as possible implantation metastasis in the event of malignancy, the decision was made, after detailed discussion with the patient and family, to proceed directly with surgical resection without prior biopsy.

Prior to surgery, specialists from the Departments of Urology and Hepatobiliary Surgery were consulted for operative planning. After multidisciplinary evaluation, it was unanimously agreed that a retroperitoneal approach would provide the least invasive and most appropriate access for complete tumor resection.

**Figure 2:**
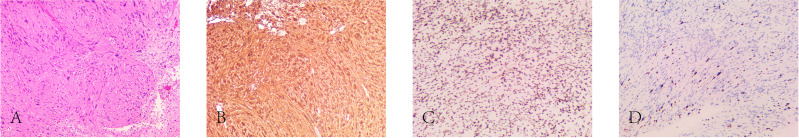
Histopathology and immunohistochemical staining of the tumor. Hematoxylin-eosin (HE) staining reveals typical histological features of schwannoma with areas of hemorrhage and cystic degeneration **(A)**. Immunohistochemical analysis shows strong positivity for S-100 **(B)**, retained nuclear expression of H3 K27me3 indicating no loss **(C)**, and a Ki-67 labeling index (LI) of approximately 10% in hotspot regions **(D)**. The tumor also demonstrates positivity for SOX-10 and Vimentin, focal weak positivity for CD34, and negativity for SMA, GFAP, Desmin, and MDM2, consistent with a diagnosis of schwannoma.

On May 29, 2025, the patient underwent laparoscopic resection of the retroperitoneal mass. The patient was positioned in the left lateral decubitus position. Following routine abdominal preparation, three laparoscopic ports were established: one along lateral border of the right rectus abdominis at the level of the umbilicus, one superomedial to the right anterior superior iliac spine, and one below the right costal margin along the midclavicular line. After entry into the peritoneal cavity, the intestines were carefully inspected, with no evidence of traumatic injury observed. Dissection was then carried out along the planes of the right kidney, adrenal gland, and inferior vena cava. A well-defined mass measuring approximately 3.0 × 2.5 cm was identified posterior to the thoracic vein, closely adherent to the right adrenal gland. The tumor was meticulously dissected free, completely resected, and the patient’s blood pressure and heart rate stabilized during the procedure. The surgery was performed without major complications, and the specimen was sent for pathological examination. The postoperative course was uneventful, with the patient showing no signs of active bleeding or infection. Histopathology confirmed a diagnosis of a schwannoma with hemorrhagic and cystic degeneration, and immunohistochemical markers supported the diagnosis of a neurogenic tumor (**Figure 2**).

The patient’s post-operative recovery was closely monitored. On June 1, 2025, a follow-up blood test revealed elevated inflammatory markers and slight liver dysfunction, but there was no significant change in renal or cardiac function. On June 2, a CT scan showed residual post-surgical fluid and minimal pleural effusion, with no evidence of tumor recurrence.

The patient was discharged on June 4, 2025, with stable vital signs and no significant discomfort. His discharge diagnoses included retroperitoneal schwannoma, hypertension, coronary artery disease, and fatty liver. He was advised to maintain a low-fat, high-protein diet and to follow up with oncology, cardiology, and hepatobiliary clinics for continued care. Additionally, surgical wound care instructions were provided, with scheduled follow-up visits for further monitoring.

In summary, this 74-year-old male presented with an incidental retroperitoneal mass and was diagnosed with a schwannoma after imaging and surgical resection. His postoperative recovery was smooth, and he was discharged with plans for ongoing surveillance.

## 3 Discussion

This case presents a 74-year-old male with a retroperitoneal mass discovered incidentally during a routine physical examination. The mass, confirmed as a schwannoma following surgical resection, represents a rare occurrence of a neurogenic tumor located in the retroperitoneal space. Schwannomas, while common in peripheral nerves, are infrequent in this anatomical region. This case highlights several aspects related to clinical diagnosis, surgical intervention, and postoperative management, contributing to a better understanding of retroperitoneal tumors in the elderly.

Schwannomas, particularly those found in the retroperitoneal space, are uncommon, accounting for only a small percentage of retroperitoneal tumors. These tumors are typically benign and slow-growing, often asymptomatic until they reach a size large enough to cause compressive effects on adjacent structures (1, 4, 5). In this case, the 3.0 x 2.5 cm mass, detected incidentally during a routine abdominal CT, is a classic example of an asymptomatic schwannoma. This emphasizes the significance of regular imaging and thorough diagnostic evaluations, even in the absence of clinical symptoms (6, 7). The incidental discovery of such masses underscores the need for careful interpretation of imaging findings and highlights the importance of monitoring such lesions when they are asymptomatic, as in this case (8).

However, there are certain limitations to this case. First, the retroperitoneal schwannoma is a rare diagnosis, and the literature on its management, especially in elderly patients, is sparse (9). The lack of a more extensive long-term follow-up in this case limits the ability to fully assess the recurrence rate or potential metastasis of schwannomas in the retroperitoneal space. Additionally, the patient’s comorbidities, including coronary artery disease and hypertension, may complicate the clinical course in some cases, although these did not seem to affect the immediate surgical outcome (4). Further studies with a larger cohort of similar cases could provide more definitive conclusions regarding the optimal surgical approach and the long-term prognosis of retroperitoneal schwannomas in elderly patients.

In the broader context of the literature, this case aligns with the general understanding that retroperitoneal schwannomas are often asymptomatic and discovered incidentally during imaging studies for unrelated conditions (10). The size and location of the tumor in this patient, along with the absence of severe symptoms like pain or dysfunction, are consistent with what is described in other cases (4). However, it differs from some reports in which larger schwannomas cause significant symptoms due to their compression of adjacent structures (10). Furthermore, the literature suggests that despite the benign nature of schwannomas, their resection can be challenging due to their proximity to vital structures such as the kidneys, adrenal glands, and major blood vessels. One of the key challenges in managing rare tumors such as retroperitoneal schwannomas is determining the appropriate surgical approach (11).

The treatment of schwannomas primarily relies on surgical resection (12). In particular, for benign retroperitoneal schwannomas, the standard therapeutic approach is typically carried out through multidisciplinary collaboration, employing nerve-sparing minimally invasive techniques under the assistance of a surgical microscope or exoscope, with the aim of achieving complete tumor removal while preserving neural function. The study conducted by Benato et al. demonstrated that complete resection was achieved in 87% of the cases (9).The study by Nenad Koruga et al. employed a staged surgical strategy, in which the neurosurgical team performed an interlaminar resection at the left L3 level using a far-lateral approach to delicately dissect the tumor while preserving neural structures as much as possible. Subsequently, the general surgery team achieved complete tumor removal through a midline laparotomy, successfully avoiding injury to vital organs. Follow-up MRI performed five months after surgery revealed no evidence of tumor recurrence (13).For cases characterized by slow growth and absence of symptoms, regular imaging follow-up may be considered; however, surgical resection remains the preferred treatment option. Interventional therapies, such as preoperative arterial embolization (14) to reduce intraoperative bleeding, as well as image-guided radiofrequency or cryoablation (15), have gradually evolved from adjunctive treatments to alternative therapeutic options, particularly suitable for patients who are inoperable or present with recurrent disease. Pharmacological therapy is currently applied primarily for postoperative pain management, as no specific or effective medications for the treatment of schwannomas have yet been identified. In this case, laparoscopic resection was successfully performed without major complications, ensuring a minimal recovery time and reducing the risk of postoperative complications. This case reaffirms the effectiveness of minimally invasive techniques in managing retroperitoneal masses, particularly when the lesion is localized and accessible. The patient’s recovery was uneventful, which aligns with reports of low morbidity following laparoscopic resection of retroperitoneal schwannomas (10). This case exemplifies the careful dissection required to achieve complete tumor removal while minimizing damage to surrounding tissues.

The rarity of this type of tumor emphasizes the need for ongoing research and discussion in the field. It is essential that future case reports of retroperitoneal schwannomas continue to be documented to further enhance our understanding of these rare lesions. Moreover, careful management of comorbidities and close postoperative monitoring are essential for achieving favorable outcomes in this patient population (16).

In conclusion, this case highlights the importance of early detection through imaging, the role of immunohistochemistry in accurate diagnosis, and the efficacy of minimally invasive surgical techniques. The management of comorbidities is critical to ensure the safety and recovery of elderly patients undergoing surgery. Furthermore, the need for long-term surveillance and ongoing research into the management of rare retroperitoneal tumors cannot be overstated. This case serves as a valuable reference for clinicians encountering similar presentations, offering insight into the diagnostic and therapeutic strategies that can be employed for optimal patient care.

## 4 Conclusion

In conclusion, this case highlights the successful management of a rare retroperitoneal schwannoma in an elderly patient, diagnosed incidentally during a routine physical examination. The tumor was resected laparoscopically with minimal complications, underscoring the effectiveness of minimally invasive techniques for retroperitoneal masses (17). Evidence-based recommendations include the importance of early detection through imaging, careful management of comorbidities, and regular postoperative monitoring for potential recurrence or complications. This case also calls for further research on long-term prognosis and optimal treatment strategies for retroperitoneal schwannomas, especially in elderly patients with multiple comorbid conditions. Such studies will enhance clinical understanding and improve patient care in similar cases.

## Data Availability

The original contributions presented in the study are included in the article/**Supplementary Material**. Further inquiries can be directed to the corresponding authors.
