# Supplementary figures and images for: Laparoscopic resection of an incidental retroperitoneal schwannoma in a 74-year-old male: a case report and literature review
Source: Front Oncol. 2025 Nov 25;15:1668644. doi: 10.3389/fonc.2025.1668644 (PMC12685626; doi:10.3389/fonc.2025.1668644)

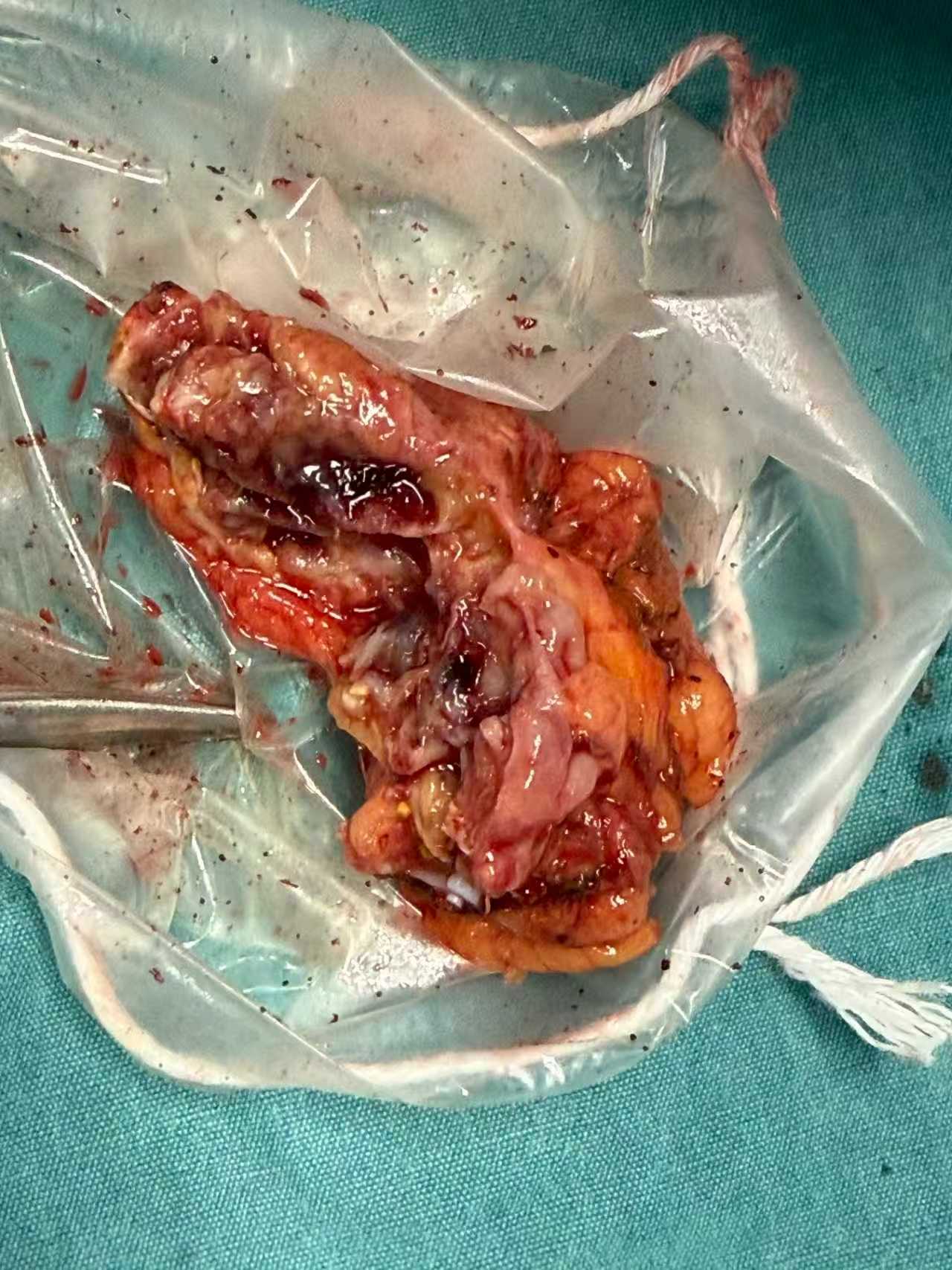

Supplement: Supplementary file 1 [file Image1.jpeg]
